# Supplementary material for: Unveiling New Genetic Variants Associated with Age at Onset in Alzheimer’s Disease and Frontotemporal Lobar Degeneration Due to C9orf72 Repeat Expansions
Source: Int J Mol Sci. 2024 Jul 7;25(13):7457. doi: 10.3390/ijms25137457 (PMC11242823; doi:10.3390/ijms25137457)
Supplement: Supplementary file 1 [file ijms-25-07457-s001.zip › Table S1, S2.pdf]

Table S1. Population frequencies of the rs1049296 *TF* and the rs7550295 *CLSTN1* variants.

**gnomAD**	**rs1049296 *TF* (Genome/Exome)**	**rs7550295 *CLSTN1* (Genome/Exome)**
Admixed American	0.148/0.123	0.132/0.190
African/African American	0.076/0.080	0.251/0.261
Amish	0.177/-	0.110/-
Ashkenazi Jewish	0.215/0.211	0.056/0.052
East Asian	0.253/0.245	0.139/0.168
European (Finnish)	0.102/0.111	0.027/0.030
European (non-Finnish)	0.159/0.163	0.049/0.049
Middle Eastern	0.201/0.218	0.048/0.067
Remaining	0.159/0.171	0.105/0.088
South Asian	0.220/0.013	0.287/0.272
Total	0.138/0.165	0.123/0.076

gnomAD, genome aggregation database.

Table S2. List of genetic features for genetic and sporadic patients.

Gene	Mutation	N. carriers (%)	Group
GRN	p.Leu271LeufsX10	37 (92.5%)	*GRN* mutation carriers
	p.Cys306X	1 (2.5%)	
	p.Gln341X	1 (2.5%)	
	p.Cys139Arg	1 (2.5%)	
**Gene**	**Repeat expansion**	**N. carriers (%)**	**Group**
C9orf72	Pathological (>30)	28 (70%)	*C9orf72* expansion carriers
	Intermediate (12-30)	12 (30%)	
**Gene**	**Genotype**	**N. carriers (%)**	**Group**
APOE	e3/e4	45.2%	sporadic AD
	e4/e4	6.5%	
